# Youth Empowered Self-Care: a direct-to-participant intervention to promote mental well-being

**DOI:** 10.1186/s12889-026-28143-0

**Published:** 2026-06-29

**Authors:** Emily M. D’Agostino, Elizabeth R. Pulgaron, Cody Neshteruk, Camille Brown-Lowery, Princess Abbot-Grimes, Tigidankay Fadika, Mark Ward, Jeannine Sato, Mia Cooper, Eric Hansen, Erin Enwright, Maria I. Nardi, Jeffrey Forde, Andrea Hicks, Tang Li, Christoph P. Hornik

**Affiliations:** 1 Duke Clinical Research Institute, Duke University School of Medicine, 311 Trent Drive, 2nd FL, Occupational Therapy Doctorate Division, Durham, NC 27710 USA; 2 Department of Orthopaedic Surgery, Duke University School of Medicine, Durham, NC USA; 3 Department of Population Health Sciences, Duke University School of Medicine, Durham, NC USA; 4 Duke Center for Childhood Obesity Research, Duke University School of Medicine, Durham, NC USA; 5 Duke Global Health Institute, Duke University School of Medicine, Durham, NC USA; 6 Mailman Center for Child Development, Miller School of Medicine, University of Miami, Miami, FL USA; 7 Miami-Dade County Department of Parks, Recreation and Open Spaces, Miami, FL USA; 8 Durham Parks and Recreation, Durham, NC USA; 9 Department of Public Health Science, Penn State College of Medicine, Hershey, PA USA; 10 Department of Pediatrics, Duke University School of Medicine, Durham, NC USA

**Keywords:** Youth, Mental well-being, Mental health, Anxiety, Social connectedness, Parks and recreation, Health equity

## Abstract

**Background:**

A crisis of youth mental health exists, disproportionately affecting minoritized and low-income youth. Park- and community-based recreation programs may promote mental well-being by offering accessible and affordable recreation in non-stigmatizing settings, with the potential to reduce mental health disparities. However, these programs are not currently being used at scale. Youth Empowered Self-Care (YES) is a mental well-being intervention intended to increase timely access to park- and community-based recreation programs among minoritized low-income youth. We evaluated YES for feasibility, acceptability, and change in participants’ psychological, mental, and social well-being and anxiety.

**Methods:**

This study was a single-arm prospective cohort pilot design. Youth aged 8–12 years were enrolled with two parks and recreation departments in Durham, North Carolina, and Miami, Florida, United States. Youth and caregivers were directed to the YES website, where they received information on mental health and resources, and park- and community-based programs. Data collected included website usage, program enrollment, and caregiver-reported youth psychological, mental, and social well-being and anxiety at baseline and at 3 months using the KIDSCREEN-27 and Screen for Child Anxiety Related Emotional Disorders (SCARED) measures to assess feasibility, acceptability, and change in psychological, mental, and social well-being and anxiety. Change from pre- to post-test scores and paired t-tests were used to determine change in mental well-being via the KIDSCREEN-27 and SCARED assessments.

**Results:**

Participants (*n* = 247) included youth aged 8–12 years (36.4% Black;34.4% White; 11.7% Hispanic; 50.2% male; 39.7% >$50,000–$75,000 annual household income). The YES website had > 1,100 users, with ≥ 51.0% of website users enrolling in YES. Almost all (99.1%) participants indicated that YES connected them to a program, and that they subsequently completed their program (99.5%). Eighty-five percent and 31.3% of caregivers reported an increase on the youth psychological well-being and peer and social support subscales, respectively. Nearly half (46.5%) of caregivers reported a reduction in youth anxiety.

**Conclusions:**

This study suggests that YES may promote youth mental well-being. Pilot data also demonstrated YES feasibility and acceptability, and linkage to mental health education and treatment. The next steps for this research entail randomized prospective trials to assess YES efficacy.

**Trial registration:**

NCT06255093 (www.clinicaltrials.gov); registration date: February 12, 2024.

**Supplementary Information:**

The online version contains supplementary material available at 10.1186/s12889-026-28143-0.

## Background

One in five children in the United States (US) are adversely impacted by a mental health condition, but less than half will receive treatment [1]. The COVID-19 pandemic exacerbated rates of mental health conditions, nearly doubling the prevalence of both anxiety (11.6% to 20.5%) and depression symptoms (12.9% to 25.2%) in youth worldwide [2], resulting in an overburdened healthcare system that prevents youth from accessing care [3, 4]. The significant gap between demand and availability of mental health resources for youth has led to increased waiting lists of 20–26 weeks in major US health systems [3]. In response to this youth mental health crisis, the American Academy of Pediatrics and US Surgeon General have declared a national state of emergency, calling for innovative strategies to screen and identify youth with mental health conditions and refer them to appropriate resources, highlighting prevention as a critical priority [5–7].

Youth disparities in access to mental health providers and resources are particularly dramatic for minoritized racial and ethnic, and socioeconomic groups. Non-Hispanic White youth have twice the likelihood (17.7%) of receiving any mental health treatment compared to Black (8.7%) and Hispanic (9.2%) youth [8]. Minoritized and low-income youth also have less access to park- and community-based recreation programs that play a critical role in supporting youth physical and mental health, and this may in part explain disparities in mental well-being [9, 10].

Park- and community-based recreation programs offer a unique opportunity for fostering psychosocial support and social connectedness [11–13] and reducing anxiety [9, 10], particularly for underserved youth [7, 14–19]. Despite these benefits, and even though parks are ubiquitous and proximal, they are underutilized assets for youth who live in under-resourced areas [20]. Children and teens account for just 20% and 7% of park users in the US, respectively, and local household poverty level was associated with a 12% decrease in park use [21]. Novel interventions that integrate diverse community prevention strategies offer solutions for equitable youth mental health, particularly among underserved populations [7, 22]. Leveraging park- and community-based recreation programs is a promising avenue to provide safe and accessible places for minoritized and low-income youth to promote mental well-being and reduce mental health disparities.

The long-standing community-engaged partnerships between the Duke University School of Medicine and both Durham Parks and Recreation (DPR, Durham, NC) and Miami-Dade County Parks, Recreation and Open Spaces (MDPROS, Miami, FL) have fostered a codesigned, co-led, park-based youth intervention called Youth Empowered Self-Care (YES; https://youandmehealthy.org/yes/). YES operates in under-resourced settings to connect minoritized and low-income youth to parks and recreation programs in order to improve mental well-being. The parks and recreation department staff act as equal partners in our study leadership team, including co-developing all participant recruitment and data collection measures, co-leading our steering committee, and co-disseminating all study findings. This study team also has extensive experience collaborating on multiple prior projects related to promoting youth health equity in parks and recreation settings [14, 23–25]. The aim of this study was to conduct a single-arm prospective cohort pilot study to evaluate the YES intervention’s feasibility, acceptability, and change in participants’ psychological, mental, and social well-being and anxiety.

## Methods

### Study design

In this pilot study, we evaluated the YES intervention over 9 months in Durham, NC, and Miami, FL, using a single-arm prospective cohort design. The study was conducted in accordance with the Declaration of Helsinki. Caregiver participants provided informed consent, and child participants provided assent. The study and all procedures were approved by the Duke University Health System Institutional Review Board (Pro00114773).

### Parks and recreation programs

The pilot study was held at two parks and recreation departments: DPR and MDPROS. DPR has been the public resource for open spaces, playgrounds, athletic programs and facilities, aquatics and all manner of recreational activity for the residents and visitors of Durham for over 100 years. DPR includes 68 parks, 56 playgrounds, and 30 miles of trails and greenways, and has extensive ties to the Durham community. MDPROS is the third largest accredited county park system in the US with 290 parks spanning 40,000 acres of land, and serves a geographic region with particularly high socioeconomic and racial/ethnic diversity. MDPROS comprises after-school and summer-camp programs, educational nature centers and conservancies, environmental restoration efforts, and arts and cultural programs and events. Within Durham and Miami, YES is offered in under-resourced areas with high social vulnerability (see Additional file 1) [26]. DPR youth programs serve historically marginalized (55% Black, 18% Hispanic) youth residing in areas with $19,000 mean per capita income, significantly below the city median ($58,190). MDPROS primarily serves minoritized youth (50% Hispanic, 47% non-Hispanic Black) residing in high or very high poverty areas (47.5% and 23%, respectively).

### Recruitment

Youth participants can learn about and enroll in the parks and recreation programs directly on the DPR and MDPROS websites. Programming runs throughout the year at up to 9 DPR and 22 MDROS sites, and participants are encouraged to sign up for multiple programs over the course of the year. All DPR and MDPROS youth programs offered through YES are led by the departments’ youth recreation staff. Programs range in cost from free to $25 per week with sliding scale options. Participants for this study were youth and their caregivers who were recruited in the first 6 months of the study on a rolling basis through direct-to-participant strategies including social media (Facebook, Twitter/X), printed flyers distributed to local recreation sites, schools and community programs/events, on-site information sessions with MDPROS and DPR staff, and word of mouth. All recruitment, data collection, and dissemination materials were offered to participants in English and Spanish. Recruitment materials informed participants about YES and invited them to visit the YES website to enroll in the study (see Additional file 2 for the pilot study design flow). Youth were eligible to participate if they were 8–12 years old at enrollment (to align with the majority of MDPROS and DPR youth programs vs. teen programs), lived in or near Durham County, NC, or Miami-Dade County, FL, and completed both consent and assent forms as well as baseline surveys. Caregivers were eligible to participate if they were a parent or legal guardian of a potential youth participant.

### Intervention 

#### YES Overview

is a multilevel mental well-being intervention that promotes access to park- and community-based recreation programs among minoritized and low-income youth. The initiative was codeveloped under the joint leadership of Duke University, DPR, and MDPROS. YES is intended to increase youth mental well-being through providing youth and families with access to an online portal. This portal helps youth and families to (1) complete validated surveys to assess youth mental well-being; (2) obtain access to online education and information about community well-being resources; and (3) become connected to park staff (YES Connector) who can provide guidance about age-appropriate programs of interest. YES is designed to target mental well-being through mechanisms grounded in Social Cognitive Theory [27], including social support and social networks. Specifically, the intervention connects youth to park- and community-based recreation programs and spaces, and addresses multiple levels (see Table 1), ranging from intra- and interpersonal factors among peers and family to organizational/recreation site influences and park-based structural factors.

**Table 1:** Overview of YES

Multiple Levels of Intervention	Dosage	Perceived Benefit Targeted
Individual: Online education and community resource linkages, and increasing youth sense of community	Ongoing	Youth mental well-being
Organizational/Recreation Center: Connection to youth programs, composition and dynamics of peers, staff/mentors present, programs, field trips and events offered	Daily or Weekly (program dependent)	Youth social connectedness, peer and social support, family functioning
Community/Environment: Parks and Recreation site facilities; Greenspace access; Community mental health resources	Ongoing	Reducing barriers to underserved youth mental well-being promotion resources

#### Individual-level components

YES is offered to individual youth through an online direct-to-participant website available in English and Spanish. The website includes online education and resources, assessments, and information on parks and recreation programs offered, along with how to reach a YES Connector (park staff member) to help select age-appropriate programs that are best suited for participants’ schedules, location preferences, and interests. The YES Connector also helps participants with enrolling in the DPR and MDPROS regularly scheduled youth programs (e.g., aquatics, athletics, fitness, outdoor recreation, cultural heritage, arts, music, cooking and other enrichment, school-age care and afterschool programs, summer camps, and special programs for youth with disabilities).

#### Organizational/recreation center–level components

The YES Connector, through providing tailored guidance to youth and families, promotes accessibility to group activities in age-appropriate parks and recreation programs, field trips and events offered by DPR and MDPROS organizations. By being connected to these programs, the youth are provided with opportunities to foster relationships among their peers and interact with recreation providers who are intended to be trusted and positive mentors typically representative of the recreation site community.

#### Environment-level components

Through YES enrollment, youth have increased access to neighborhood recreation and mental health resources that will ultimately serve to promote mental well-being. For example, YES promotes accessibility to greenspaces through programs that include outdoor recreation, nature hikes, field trips to conservancies, and nature photography. YES also connects youth to community-based mental health resources including behavioral health specialists and youth groups that focus on social skills, conflict resolution, interpersonal relationships, anxiety, health promotion, divorce support, and art therapy/expressive arts.

### Data collection

All youth and caregivers who accessed the YES website were invited to complete an eligibility screening survey, which was made available through hyperlinks provided via social media, and also through a QR code on printed flyers. If inclusion criteria were met, participants were directed to an electronic informed consent and assent form for all study components, followed by baseline surveys. These surveys included questions about youth participant demographics (see Additional file 3), prior engagement with parks and recreation (see Additional file 4), and current mental well-being (psychological, mental, and social health and well-being and anxiety; see below for details). Follow-up surveys at 3 months post-baseline were also made available through a hyperlink that was emailed to caregivers and also measured mental well-being, in addition to program satisfaction, and barriers to program participation. All consent and assent forms and surveys were completed in REDCap.

### Outcomes and endpoints

#### YES feasibility and acceptability

We assessed YES feasibility and acceptability over the 9-month study based on enrollment (targeting 150 total youth across both counties), successful completion of surveys, and connections for youth to the YES connector. The target sample size was determined based on appropriateness to understand the feasibility of YES participant recruitment and acceptability, assess descriptive change in psychological, mental, and social well-being and anxeity, and also based on anticipated dropout rate for direct-to-participant studies. We gathered data based on percentage of enrollees already enrolled in parks and recreation programs at baseline, parks and recreation program enrollment and completion, program satisfaction, and parents’ perceptions of their child’s mood after participating in the program. In addition, we assessed feasibility and acceptability based on community partner engagement by tracking frequency and attendance of YES project planning meetings, and YES website views and unique users. Pre-specified criteria included regular frequency (e.g., biweekly) planning meetings with majority of team members (> 50%) in attendance, as well as ≥ 150 YES website views and unique users (based on the target enrollment).

#### Mental well-being

Psychological, mental, and social health and well-being was assessed using the parent version of the KIDSCREEN-27 [28, 29], which is a health-related quality of life measure that assesses the subjective psychological, mental, and social health and well-being of children and adolescents. The KIDSCREEN has been validated for racial/ethnic minoritized and low-income youth ages 8 and 18 (e.g., correlations between other health related quality of life measures: *r* = .36 to 0.63), and has shown good test-retest reliability (e.g., ICC range across subscales: 0.71 to 0.78) [28–31]. The KIDSCREEN-27 assesses well-being across five subscales: Psychological Well-Being (7 items), Physical Well-Being (5 items), Autonomy and Parent Relations (7 items), Peers and Social Support (4 items), and School Environment (4 items) [28].

We measured anxiety as one of the primary outcomes for this prevention study given that it is the most prominent mental health condition in youth [32]. Anxiety was assessed using the parent version of the SCARED measure [33–35]. The SCARED measure (41 items) is widely used to assess childhood anxiety based on parent report and has been tested across clinical samples for reliability (ICCs: 0.70 to 0.90), internal consistency (α = 0.74 to 0.93), and discriminative validity (differentiated children with anxiety/nonanxiety psychiatric disorders, *p* < .005) as a screening tool for anxiety disorders (somatic/panic, generalized anxiety, separation anxiety, social phobia, and school phobia) among clinically referred children and adolescents [33–35].

#### Sociodemographic measures

Sociodemographic measures were collected at baseline, including parent-reported child gender, race, ethnicity, parent education, and household income. Age (within ages 8–12) was collected as a screening variable at enrollment to ensure youth inclusion criteria were met.

#### Program data

We assessed program type (physical activity, mixed with some physical activity, and enrichment [not involving physical activity, e.g., arts, music, educational]), size (< 10 or > 10 youth), and total sessions that each participant attended to determine total contact hours at the participant level. These program data were collected throughout the study period.

### Statistical analysis

We calculated basic descriptive statistics (i.e., means and standard deviations [SDs] for continuous variables, and frequencies and percentages for categorial variables) on demographics, survey, and process data. Chi-square and Fisher’s exact tests were performed to assess similarities and differences in descriptive (participant demographic and program) factors across counties. Chi-square tests were used if ≤ 20% of expected cell counts were < 5 (race, Hispanic ethnicity, male gender, group size, program length, and program type); Fisher’s exact test was used if > 20% of expected cell counts were < 5 (highest education, household income, reached out to YES Connector, completed program, and program duration). Descriptive mean (SD) change from pre- to post-test scores on the KIDSCREEN and SCARED assessments was calculated (percentage change = [prescore value – {postscore value/prescore value}] × 100) to determine change in mental well-being based on aggregate and individual child-level data, as well as stratified by sociodemographic (e.g., race/ethnicity, gender, income) factors. Across both assessments, paired t-tests were used to compare differences in pre- and post-tests for individual youth participants at an alpha level of 0.05. To compare mean individual change in pre-post tests across demographic factors, independent t-tests were performed for group variables with two levels, and analyses of variance were performed for group variables with more than two levels. Analyses were conducted using SAS 9.4 (SAS Institute, Cary NC), SPSS 29.0.2.0 (IBM Corp, Chicago, IL) and R version 4.3.1 (R Foundation for Statistical Computing, Vienna, Austria) via RStudio 2023.06.0 Build 421 (Posit Software, Boston, MA).

## Results

### YES feasibility and acceptability

**Fig. 1:**
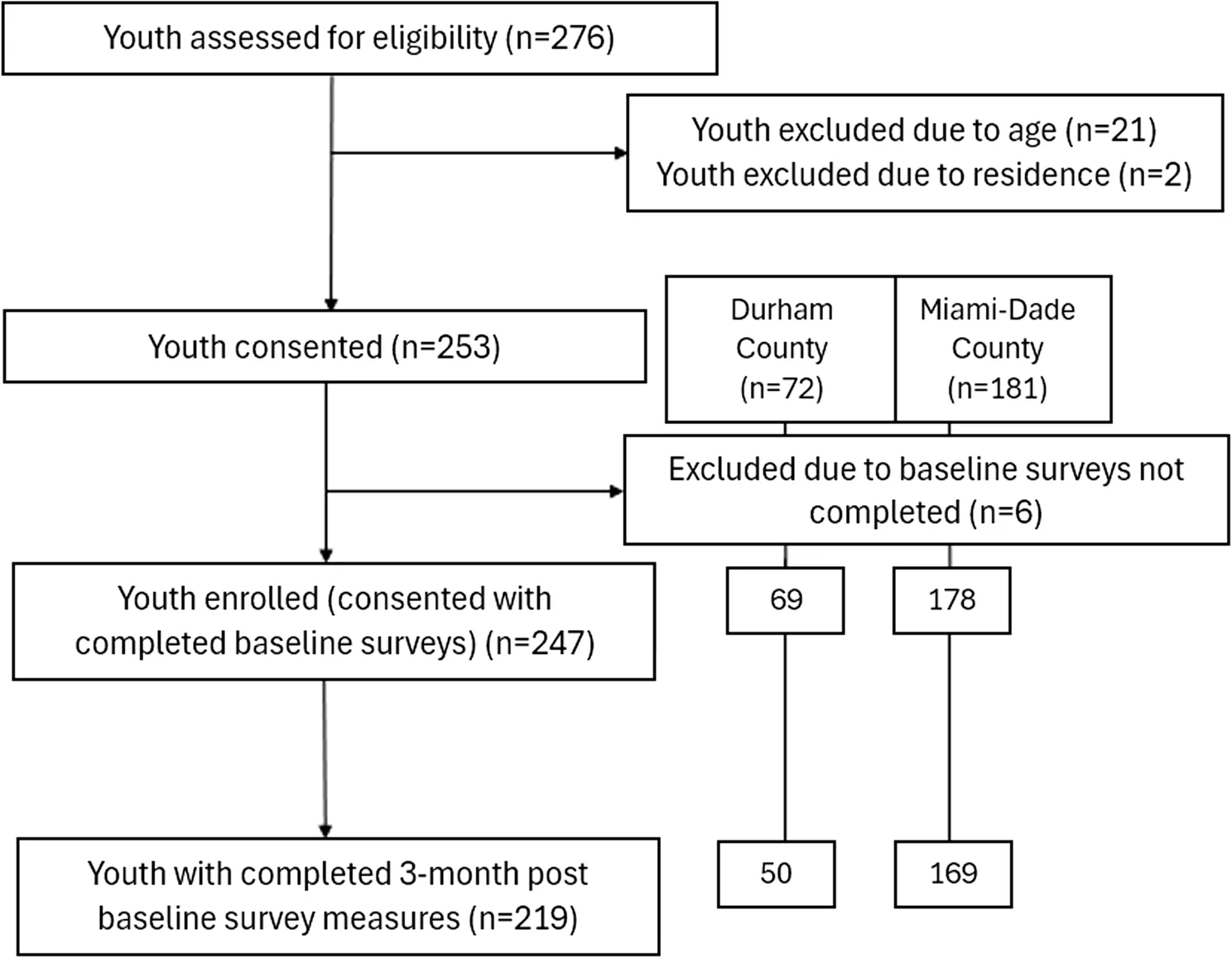
Participant flow diagram

We enrolled 247 participants ages 8–12 years (36.4% Black [*n* = 90]; 34.4% White [*n* = 85]; 11.7% [*n* = 29] Hispanic; 50.2% male [*n* = 124]; annual household income: 39.7% >$50,000–$75,000 [*n* = 98]; parents’ highest level of education: 40.5% high school or GED [*n* = 100]; 72.1% in Miami-Dade County [*n* = 178] and 27.9% in Durham County [*n* = 69]; see Table 2). We assessed 276 youth for eligibility and excluded 23 youth (ineligible due to age [*n* = 21]; place of residence [*n* = 2]). Another 6 youth did not complete baseline surveys and were therefore also excluded (see Fig. 1 for participant flow diagram ). Chi-square tests showed that counties were not statistically significantly different across all descriptive factors except for race and highest education, justifying pooling of analyses across counties. Baseline survey data indicated that the majority of youth (87.7%, *n* = 217) were not enrolled in a parks and recreation program at the time of YES enrollment. Based on the 3-month survey data (*n* = 219), almost all (99.1%, *n* = 215) caregivers indicated that YES connected their child to a program and that they completed their program (99.5%, *n* = 218). Most caregivers were satisfied (65.3%, *n* = 143) or very satisfied (33.3%, *n* = 73) with the program, and most agreed (50.2%, *n* = 110) or strongly agreed (44.8%, *n* = 98) that their child “seemed to be in a better mood after participating in the program” (see Additional file 4 for survey). Community partner engagement was maintained throughout the 9 month study with biweekly hour-long meetings with study team members (involving 9 people on average, or 64.3%; SD = 3), including up to nine Duke, three DPR, and two MDPROS staff. The YES website had 1,107 views and 482 unique users, and the mental health resources page was the most frequently visited page. Website views spiked during the enrollment period. At least 51.0% of website users enrolled in YES.

**Table 2:** YES participant characteristics

Participant and Program Characteristics	Overall (*n* = 247)		Miami-Dade County (*n* = 178)		Durham County (*n* = 69)		*p*-value^b, c^
	*n* ^a^	%	*n*	%	*n*	%	
Race							0.003
Black	90	36.4	61	34.3	29	42.0	
White	85	34.4	63	35.4	22	31.9	
Asian	55	22.3	47	26.4	8	11.6	
Other^d^	17	6.9	7	3.9	10	14.5	
Hispanic ethnicity	29	11.7	22	12.4	7	10.1	0.791
Male gender	124	50.2	91	51.1	33	47.8	0.747
Caregiver highest education							< 0.001
High school graduate or GED	100	40.5	80	44.9	20	29.0	
Associate’s degree or equivalent; some college	47	19.0	30	16.9	17	24.6	
Bachelor’s degree or equivalent	80	32.4	64	36.0	16	23.2	
Graduate degree	18	7.3	3	1.7	15	21.7	
Prefer not to answer	2	0.8	1	0.6	1	1.4	
Household income							0.382
$25–50 K	11	4.5	7	3.9	4	5.8	
>$50–75 K	98	39.7	77	43.3	21	30.4	
>$75–100 K	82	33.2	57	32.0	25	36.2	
>$100K	43	17.4	28	15.7	15	21.8	
Prefer not to answer	13	5.3	9	5.1	4	5.8	
Reached out to YES Connector	217	99.1	168	99.4	49	98.0	0.405
Completed program	215	99.5	167	100.0	48	98.0	0.227
Group size							0.443
≥ 10 youth	41	18.7	34	20.1	7	14.0	
< 10	178	81.3	135	79.9	43	86.0	
Program duration							0.099
1–3 h	190	86.8	143	84.6	47	94.0	
> 3 h	29	13.2	26	15.4	3	6.0	
Program length							0.146
1–3 weeks or 1 time	187	85.4	148	87.6	39	78.0	
4–6 weeks	32	14.6	21	12.4	11	22.0	
Program type							0.612
Physical activity	110	50.2	85	50.3	25	50.0	
Other (arts, music, educational)	74	33.8	55	32.5	19	38.0	
Mixed	35	16.0	29	17.2	6	12.0	

^a^N missing, reached out to YES Connector = 28, completed program = 31, group size = 28, program duration = 28, program length = 28, program type = 28

^b^Chi-square test used if ≤ 20% of expected cell counts < 5: race, Hispanic ethnicity, male gender, group size, program length, program type

^c^Fisher’s exact test used if > 20% of expected cell counts < 5: highest education, household income, reached out to YES Connector, completed program, program duration

^d^Other Race = American Indian/Alaska Native, Pacific Islander, Two or more races

### Program data findings

Half of participants (50.2%, *n* = 110) enrolled in a physical activity program, and 33.8% (*n* = 74) enrolled in other programs (arts and crafts, music, educational). The remaining 16.0% (*n* = 35) enrolled in programs that included a combination of physical activity and other programming. Most (86.8%, *n* = 190) enrolled in a program that was 1–3 h per session, 85.4% (*n* = 187) in a program that was either 1–3 weeks in duration or one session (85.4%, *n* = 187), and 81.3% (*n* = 178) in a program that had < 10 youth.

### Psychological, mental, and social well-being findings

#### KIDSCREEN measure

##### Psychological well-being subscale 

Participation in the YES program was associated with significant improvements in psychological well-being. Across all participants, psychological well-being increased significantly, t[212] = -16.74, *p* < 0.001. Gender differences emerged, with males reporting greater improvements (M = 3.36, SD = 2.57) compared to those reported by females (M = 2.55, SD = 2.56, F[1, 211] = 2.31, *p* = 0.022). Differences among ethnic and racial groups were significant. Hispanic youth demonstrated significantly greater gains (M = 3.96, SD = 1.86) than non-Hispanic youth (M = 2.84, SD = 2.65, t[211] = -2.04, *p* = 0.021). There were also racial differences (F[3, 208] = 5.90, *p *< 0.001), education-level differences (F[4, 208] = 4.92, *p *< 0.001), and household income-level differences (F[4, 208] = 4.69, *p *< 0.001), in reported changes in psychological well-being. No county-level differences were found. See the supplemental tables in Additional file 5 for details on Tukey’s post hoc analyses for race, education, and income variables.

##### Physical well-being subscale 

Significant changes in physical well-being were also reported overall, t[212] = 3.57, *p* < 0.001. Gender effects indicated that females (M = -1.12, SD = 2.46) reported greater decreases in physical well-being compared to males (M = -0.22, SD = 2.79, F[1, 213] = 2.49, *p* = 0.014). Differences among racial groups (F[3, 204] = 18.39, *p* < 0.001) and education levels (F[4, 208] = 14.56, *p* < 0.001) were significant. See the supplemental tables in Additional file 5 for details on Tukey’s post hoc analyses. Differences among income groups were also prevalent (F[4, 208] = 13.29, *p* < 0.001), with lower income youth improving and higher income groups declining. No county differences were found.

##### Autonomy and parent relations subscale 

Changes in autonomy and parent relations were not statistically significant overall, t[214] = 0.18, *p *= 0.859. However, gender differences were present, with males reporting improvements (M = 0.57, SD = 3.42) versus females who reported declines (M = -0.69, SD = 3.39, F(1, 213) = 2.71, *p* = 0.007). Racial differences were also significant (F[3, 206] = 17.59, *p* < 0.001), with Black youth (M = 1.69, SD = 2.56) showing improvements while White youth (M = -1.95, SD = 3.49) and those who identified as Other (M = -1.33, SD = 3.57) reported declines. Differences by ethnicity were also significant (t[214] = -3.61, *p* < 0.001), with Hispanic youth reporting improvements (M = 2.24, SD = 2.59) and non-Hispanic youth reporting declines (M = -0.34, SD = 3.44). Education level was significant (F[4, 210] = 18.30, *p* < 0.001), with youth of parents with high school degrees improving the most (M = 1.72, SD = 2.65). Income group was also significantly different (F[4, 210] = 9.72, *p* < 0.001), showing greater gains among lower income youth. Again, no county-level differences were found. See the supplemental tables in Additional file 5 for details on Tukey’s post hoc analyses.

##### Peers and social support subscale 

Changes in peer and social support were not statistically significant overall, t[216] = -1.75, *p* = 0.081. However, there were significant differences among subgroups. Specifically, males showed improvements (M = 0.47, SD = 1.57), whereas female participants reported small declines (M = -0.08, SD = 1.72), F[1, 215] = 2.48, *p* = 0.014. Race differences were significant (F[3, 209] = 10.33, *p* < 0.001), with improvements being reported for Black youth (M = 0.83, SD = 1.36) and declines being reported for White youth (M = -0.56, SD = 1.56). Hispanic youth (M = 1.12, SD = 1.36) reported more positive changes compared with those reported among non-Hispanic youth (M = 0.08, SD = 1.67, t[215] = -3.00, *p* = 0.003). Education (F[4, 213] = 10.53, *p* < 0.001) and income differences (F[4, 213] = 9.47, *p* < 0.001) were also significant, again showing more gains among lower education and lower income groups. Details are provided in the supplemental tables in Additional file 5. No county-level effects were observed.

##### School environment subscale 

Changes in school environment scores were not significant overall, t[217] = 1.23, *p* = 0.218. There were also no differences in changes across genders or county. However, there were differences across racial groups on reported changes in school environment (F[3, 209] = 16.70, *p* < 0.001), with caretakers of Black youth (M = 0.77, SD = 1.47) reporting improvements while caregivers of White youth reported reductions (M = -1.36, SD = 2.01). Caregivers of Hispanic youth reported significantly greater improvements compared to those reported for caregivers of non-Hispanic youth, t(217) = -3.12, *p* = 0.022. Significant differences were also noted across education and income groups (F[4, 213] = 15.00, *p* < 0.001; F[4, 213] = 11.40, *p* < 0.001), with lower education and lower income youth showing greater improvements. See the supplemental tables in Additional file 5 for post hoc analyses.

### Anxiety findings

#### SCARED measure

**Fig. 2:**
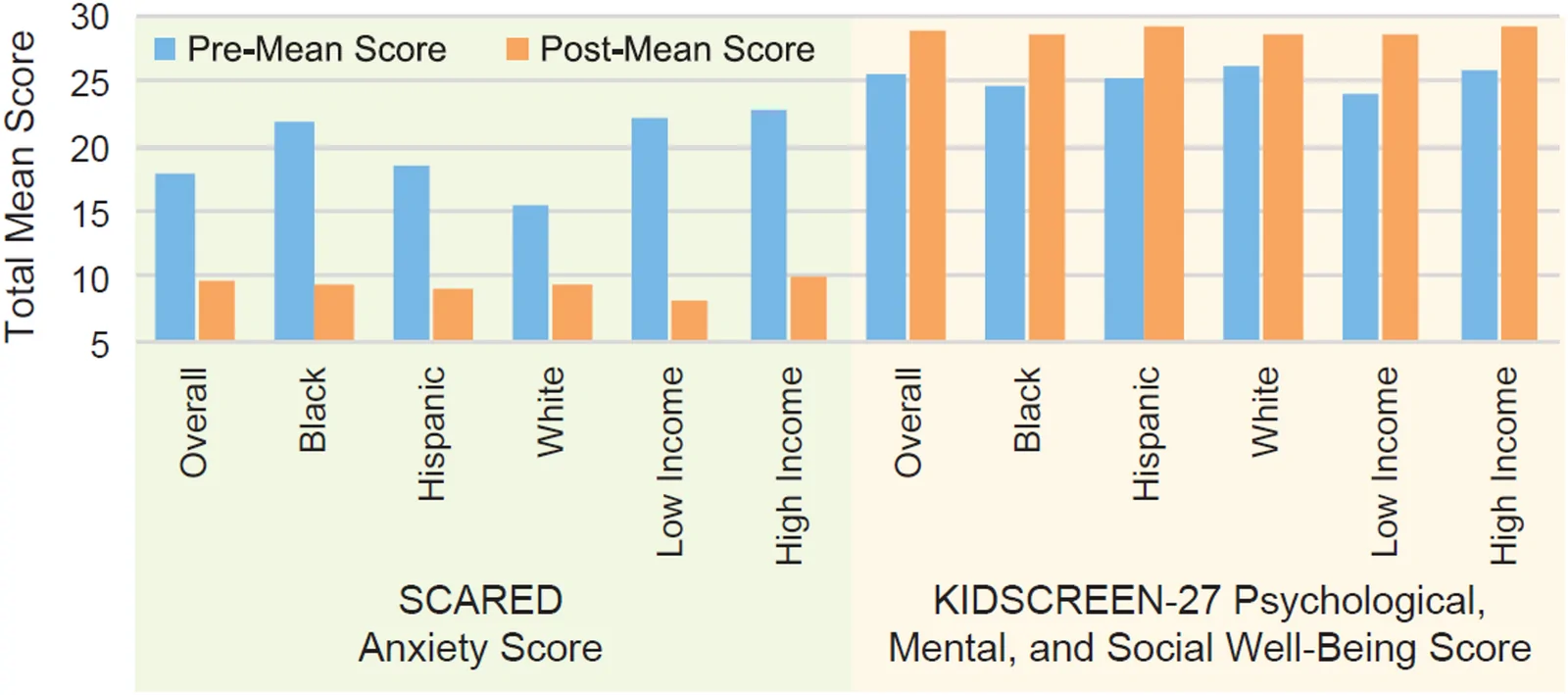
Changes in YES participant well-being overall and by race, ethnicity and income

Caregivers reported a 46.5% reduction in anxiety (t[218] = 16.72, *p *< 0.001; see Table 3; Fig. 2). Change in anxiety score was significantly different across education (F[4, 214] = 9.84, *p *< 0.001), income (F[4, 214] = 11.29, *p *< 0.001), ethnicity (t[218] = -3.22, *p*=.003), and race (F[3, 215] = 7.75, *p *< 0.001). Caregivers with a bachelor’s degree or equivalent showed youth with smaller reductions in anxiety compared to other education groups (see Table 3). A greater reduction in anxiety score was reported by caregivers in lower compared with higher income categories, caregivers of Hispanic compared with Non-Hispanic children, and also Black children compared with other race groups. Change in anxiety scores did not differ by county or gender (*p* = 0.482 and *p* = 0.325, respectively).

**Table 3:** Change in SCARED and KIDSCREEN-27 Assessment Scores Pre- and Post-YES Participation

				KIDSCREEN-27														
	SCARED (*N* = 219)			Psychological Well-Being (*N* = 213)			Physical Well-Being (*N* = 213)			Autonomy and Parent Relations (*N* = 215)			Peers and Social Support (*N* = 217)			School Environment (*N* = 218)		
	Mean change in test score (SD)^a^	t/F value^b, c^	*p*-value	Mean change in test score (SD)	t/F value	*p*-value	Mean change in test score (SD)	t/F value	*p*-value	Mean change in test score (SD)	t/F value	*p*-value	Mean change in test score (SD)	t/F value	*p*-value	Mean change in test score (SD)	t/F value	*p*-value
Overall	-5.14 (4.55)	16.72	< 0.001	2.97 (2.59)	-16.74	< 0.001	-0.65 (2.67)	3.57	< 0.001	-0.04 (3.45)	0.18	0.859	0.20 (1.66)	-1.75	0.081	-0.17 (2.08)	1.23	0.218
County		-0.71	0.482		0.17	0.867		-0.73	0.466		0.52	0.603		-0.30	0.765		0.11	0.910
Miami-Dade	-5.01 (4.25)			2.99 (2.54)			− 0.73 (2.60)			0.02 (3.41)			0.18 (1.74)			-0.17 (2.13)		
Durham	-5.60 (5.48)			2.92 (2.80)			-0.41 (2.91)			-0.27 (3.62)			0.26 (1.40)			-0.20 (1.93)		
Gender		-0.99	0.325		2.31	0.022		2.49	0.014		2.71	0.007		2.48	0.014		1.86	0.064
Male	-5.44 (4.78)			3.36 (2.57)			-0.22 (2.79)			0.57 (3.42)			0.47 (1.57)			0.08 (2.05)		
Female	-4.83 (4.29)			2.55 (2.56)			-1.12 (2.46)			-0.69 (3.39)			-0.08 (1.72)			-0.44 (2.09)		
Race		7.75	< 0.001		5.90	< 0.001		18.39	< 0.001		17.59	< 0.001		10.33	< 0.001		16.7	< 0.001
Black	-6.88 (4.38)			3.65 (2.55)			0.81 (2.55)			1.69 (2.56)			0.83 (1.36)			0.77 (1.47)		
White	-3.92 (4.61)			1.99 (2.64)			-2.04 (2.36)			-1.95 (3.49)			-0.56 (1.56)			-1.36 (2.01)		
Asian	-4.74 (4.22)			3.33 (2.40)			-0.54 (2.26)			0.27 (3.22)			0.42 (1.87)			0.17 (2.25)		
Other	-2.00 (2.35)			3.11 (1.76)			-2.00 (1.91)			-1.33 (3.57)			0.22 (1.20)			-0.44 (1.94)		
Ethnicity		-3.22	0.003		-2.04	0.021		-2.61	0.010		-3.61	< 0.001		-3.00	0.003		-3.12	0.022
Hispanic	-8.12 (5.09)			3.96 (1.86)			-0.82 (2.65)			2.24 (2.59)			1.12 (1.36)			1.00 (1.67)		
Non-Hispanic	-4.74 (4.33)			2.84 (2.65)			0.64 (2.48)			-0.34 (3.44)			0.08 (1.67)			-0.33 (2.09)		
Highest education		9.84	< 0.001		4.92	< 0.001		14.56	< 0.001		18.3	< 0.001		10.53	< 0.001		15	< 0.001
High school graduate or GED	-6.37 (4.28)			3.51 (2.52)			0.53 (2.44)			1.72 (2.65)			0.74 (1.37)			1.50 (0.15)		
Associate’s degree or equivalent; some college	-5.52 (3.91)			3.53 (2.49)			-0.76 (2.26)			-0.50 (3.19)			0.29 (1.52)			2.07 (0.32)		
Bachelor’s degree or equivalent	-2.81 (3.38)			1.99 (2.42)			-2.27 (2.13)			2.14 (3.23)			-0.40 (1.68)			-1.54 (2.00)		
Graduate degree	-6.40 (7.80)			1.20 (3.35)			-0.60 (4.77)			-2.00 (4.06)			-2.80 (2.68)			-1.00 (3.81)		
Prefer not to answer	-14.00 (18.38)			3.50 (2.12)			2.00 (2.83)			3.50 (4.95)			0.00 (0.00)			0.50 (2.12)		
Household income		11.29	< 0.001		4.69	0.001		13.29	< 0.001		9.72	< 0.001		9.47	< 0.001		11.4	< 0.001
$25–50 K	-9.88 (3.23)			4.75 (2.43)			3.25 (3.28)			3.29 (2.14)			1.38 (1.06)			1.62 (1.30)		
>$50–75 K	-6.14 (4.58)			3.48 (2.57)			-0.13 (2.52)			0.82 (3.30)			0.52 (1.44)			0.23 (1.75)		
>$75–100 K	-4.18 (3.48)			2.49 (2.45)			-1.12 (2.11)			-0.69 (3.22)			0.06 (1.29)			-0.45 (1.86)		
>$100K	-2.27 (3.56)			1.94 (2.67)			-2.45 (2.58)			-2.27 (2.91)			-1.03 (2.24)			-1.70 (2.51)		
Prefer not to answer	-9.30 (7.09)			4.33 (1.58)			1.30 (2.16)			2.40 (3.66)			1.67 (1.87)			1.90 (1.73)		

*SCARED *Screen for Child Anxiety Related Emotional Disorders, *SD *standard deviation

^a^Mean percentage individual change in test score = postscore – prescore

^b^Paired t-tests were performed for overall change in SCARED and KIDSCREEN-27 assessment scores pre- and post-YES participation

^c^Independent t-tests were performed for group variables with 2 levels; analyses of variance were performed for group variables with more than 2 levels

## Discussion

We successfully completed a pilot study of YES, a mental well-being intervention that is intended to increase timely access to park- and community-based recreation programs among minoritized low-income youth. These pilot data demonstrate successful delivery of the YES intervention in two areas that serve minoritized low-income youth, with potential for translation to other urban communities with underserved populations. We demonstrated the feasibility in recruitment, retention, community engagement, data collection, and delivery. We also demonstrated YES acceptability to promote youth mental well-being in two different parks and recreation settings. Importantly, these pilot data demonstrate that psychological, mental, and social well-being and anxiety improved for YES participants. The next steps for this research include conducting an individually randomized crossover prospective trial to assess YES efficacy, the role of social connectedness as the mechanism of effect, and optimal intervention dosage.

To address mental health needs, prevention must be prioritized, and novel approaches and settings must be used [7, 36]. The status quo as it pertains to improving youth mental well-being has been to target individual-level factors such as coping skills, problem-solving abilities, and communication capability [37, 38]. However, youth mental well-being is affected through a complex interaction of individual, interpersonal, organizational, and community factors, such as social support from peers and family [39–42], and neighborhood access to recreation and psychosocial resources [7, 14–17, 19]. Interventions that integrate diverse community prevention strategies and offer preventive solutions to reduce youth mental health disparities must be prioritized.

There is a critical need to close mental health equity gaps via programs that increase minoritized and low-income youth mental well-being by improving access to park- and community-based recreation programs. The YES intervention leveraged parks and recreation as a setting for promoting youth mental well-being. Over 108,000 public parks are managed by about 9,000 local parks and recreation departments and organizations in the US. More than 75% of these parks offer sports programming, 88% offer social recreation, 62% offer visual or performing arts, 65% offer teen programs, and more than half offer after school youth programs [43]. Prior research shows that park- and community-based recreation programs provide violence prevention and resilience promotion programming for youth. For example, the Fit2Lead study of a park-based program demonstrated that parks and recreation settings offer substantial opportunity to mitigate risk, prevent juvenile crime, build resilience, and disrupt downward mental well-being trajectories for youth during early adolescence [15]. Specifically, youth with more overall adversity reported significant improvements in self-efficacy to resolve peer conflict [17]. Additional research has shown that 40%–50% of youth at the highest mental health risk improved their social skills and problem behaviors after participating in park-based recreation programs [44].

In our study, we observed a 46.5% reduction in youth anxiety, indicating a clinically meaningful change based on previous literature [45, 46]. Importantly, observed change in anxiety scores did not appear to differ by county, indicating potential for generalizability to other geographic regions and settings. However, additional research is needed to assess the full potential of parks and recreation to promote youth mental well-being using randomized comprehensive studies that rigorously evaluate the efficacy of such a program and examine the mechanism of action. This work will help to inform public health interventions at sufficient scale to address the youth mental health crisis with preventive approaches at the population level.

Underserved youth may derive the greatest potential from park- and community-based recreation programs, given that health care providers and schools are insufficient to support youth in meeting mental health needs, and park- and community-based recreation programs are widely accessible and affordable [15, 43, 47–49]. Minoritized and low-income youth are more likely to face barriers to receiving support from mental health providers, and these barriers increase risk of negative mental health trajectories and widen mental health disparities. Park- and community-based recreation programs can provide connections to resources to help mitigate the stigma associated with seeking mental health care by promoting these services in their settings. In this study, we leveraged parks and recreation, a novel yet readily accessible resource for youth mental well-being programs and spaces to engage in healthy activity. We retained 86% of youth pre-post assessment for all measures, and observed greater improvements in mental well-being, including greater psychological well-being and peer and social support, and lower anxiety for racial/ethnic minoritized (Black and Hispanic) youth and those living in lower income households. By leveraging park-based programming to promote youth mental well-being, YES presents a novel approach for addressing mental health equity gaps at a population level, and has potential for a wider impact to address disproportionately high mental health and anxiety needs among minoritized and low-income youth [7, 22].

This study has several important limitations. First, we administered parent report measures to assess changes in youth well-being. We are unaware, however, if all parent reports were completed by biological parents or other caregivers, such as step-parents, family guardians such as grandmother or aunt/uncle, and so forth. Also, although these parent measures have been validated for racial/ethnic minoritized and low-income youth and have shown good test-retest reliability [29–31, 50], future studies should collect both parent- and youth-reported measures to allow for data triangulation where investigators can ask youth in order to quasi-validate but also compare with adult measures. Also, this study design did not include randomization or have a comparison group; therefore, causal effects cannot be inferred. Moreover, the study may have suffered from response bias and social desirability bias. In addition, this pilot study was exploratory in nature; therefore a large number of constructs were considered and analyses conducted. As a result, we recognize the potential for type 1 error and plan to apply these initial findings to help determine which factors a program like YES might impact to inform future analyses. We also did not collect caregiver demographic data. Future studies should include this information to more comprehensively describe the study population and determine whether caregiver demographics may be associated with youth study outcomes. Finally, our findings may not be generalizable to other communities and parks and recreation organizations. Although two sites were included in this study and similar results were found, future work should assess YES efficacy in other areas for wider dissemination and implementation, such as through national parks and recreation channels.

## Conclusion

Our pilot data demonstrate YES feasibility and acceptability, as well as its linkage to mental health education and treatment, and improvement in psychological, mental, and social well-being and anxiety. This work may support future YES testing and implementation in parks and recreation programs and settings where youth mental well-being can be fostered. The next steps for this research include assessing YES efficacy, identifying the mechanism of action and potential effect modifiers of YES, and determining optimal intervention dosage.

## Supplementary Information

Additional file 1. CDC’s Social Vulnerability Index Map.

Additional file 2. YES Pilot Study Design Flow.

Additional file 3. Demographic Survey.

Additional file 4. Program Satisfaction Survey.

Additional file 5. Supplemental Tables.

## Data Availability

The datasets used and/or analyzed during the current study are available from the corresponding author on reasonable request.

## **Abbreviations**

DPR: Durham Parks and Recreation
MDPROS: Miami-Dade County Parks, Recreation and Open Spaces
YES: Youth Empowered Self-Care.
