# Supplementary material for: Youth Empowered Self-Care: a direct-to-participant intervention to promote mental well-being
Source: BMC Public Health. 2026 Jun 29;26:2403. doi: 10.1186/s12889-026-28143-0 (PMC13491775; doi:10.1186/s12889-026-28143-0)
Supplement: Supplementary file 1 [file 12889_2026_28143_MOESM1_ESM.docx]

CDC’s Social Vulnerability Index Map

Source: Centers for Disease Control and Prevention; Agency for Toxic Substances and Disease Registry; Geospatial Research, Analysis, and Services Program. CDC/ATSDR Social Vulnerability Index 2020 Database U.S. Available at: https://www.atsdr.cdc.gov/placeandhealth/svi/data_documentation_download.html.
