# Supplementary material for: Youth Empowered Self-Care: a direct-to-participant intervention to promote mental well-being
Source: BMC Public Health. 2026 Jun 29;26:2403. doi: 10.1186/s12889-026-28143-0 (PMC13491775; doi:10.1186/s12889-026-28143-0)
Supplement: Supplementary file 3 [file 12889_2026_28143_MOESM3_ESM.docx]

| Eligibility Section  This information will tell us if you are able to participate in the study | |
| --- | --- |
| Please select the county you live in. | a. Durham County, NC b. Miami-Dade County, FL c. I **do not** live in Durham County or Miami-Dade County |
| What is your zip code? |  |
| What is your child’s age? | a. 7 or younger b. 8 to 12 c. 13 or older d. I do not have any children |

| Demographic Section  Please tell us a little more about you |  |
| --- | --- |
| What is your first & last name? |  |
| What is your email address? |  |
| What is your phone number? |  |
| What is your highest level of educational attainment? | a. Less than high school  b. High school graduate or GED  c. Some college or vocational  d. Associates degree or equivalent  e. Bachelor’s degree or equivalent  f. Master’s degree or equivalent  g. Doctoral degree or equivalent  h. Prefer not to answer |
| What is your current employment status? | a. Employed, full-time  b. Employed, part-time  c. Not employed, looking for work  d. Not employed, not looking for work  e. Retired  f. Disabled, not able to work  g. Prefer not to answer |
| What category best represents your total annual household income before taxes: | a. Less than $25,000  b. Between $25,000 and $50,000  c. Between $50,000 and $75,000  d. Between $75,000 and $100,000  e. Between $100,000 and $150,000  f. Between $150,000 and $200,000  g. Greater than $200,000  h. Prefer not to answer |
| Please tell us a little more about your child | |
| What is your child’s first and last name? |  |
| What grade is your child in? | a. 1^st^  b.2nd  c. 3rd  d. 4th  e. 5th  f. 6th |
| With which gender does your child most identify? | a. Man  b. Woman  c. Transgender  d. Non-binary  e. Other, please specify: _________________________________  f. Prefer not to answer |
| What is your child’s race? | a. White b. Black/African American c. Asian d. American Indian/Alaskan Native e. Pacific Islander f. Two or more races g. Other, please specify:________________ h. Prefer not to answer |
| What is your child’s ethnicity? | a. Hispanic b. Not Hispanic c. Prefer not to answer |
