# Supplementary material for: Youth Empowered Self-Care: a direct-to-participant intervention to promote mental well-being
Source: BMC Public Health. 2026 Jun 29;26:2403. doi: 10.1186/s12889-026-28143-0 (PMC13491775; doi:10.1186/s12889-026-28143-0)
Supplement: Supplementary file 4 [file 12889_2026_28143_MOESM4_ESM.docx]

| Tell us about your child’s Parks And Recreation program participation after enrolling in the YES Study | |
| --- | --- |
| How did you learn about the YES Substudy | a. I visited the YES Study website/internet b. I saw a YES study flyer (at school, parks and recreation office or Community event, etc.) c. I am a member of my local Parks and Recreation program d. I attended a Community Event e. I saw a post on Social media f. I was recommended by a friend/colleague g. Other (etc.) |
| Has your child participated or is currently participating in a parks and recreation program offered by Miami-Dade or Durham Parks and Recreation? | a. Yes  b. No  *Require a response to this question* |
| Was your child actively participating in a Parks and Recreation program at the time of your enrollment in the YES Study? | a. Yes  b. No  *Require a response to this question* |
| ***For participants that select Yes*** *transition to the “Tell us about the program your child participated in”. From here, the participants must complete the KIDSCREEN and SCARED assessments again.* | ***For participants that select No*** *transition to the “Tell us about your barriers to participation” section and then to the end of the survey* |
| Tell us about the program(s) your child has participated in while enrolled in the YES Study.  If your child enrolled in multiple programs please answer about each program | |
| Did you select a program(s) recommended by your designated YES Connector? | a. Yes b. No c. Did not reach out to YES Connector |
| What type of program(s) did your child participate in?  (please select all that apply) | d. Individual e. Small groups (less than 10) f. Large groups (more than 10) g. Indoor h. Outdoor |
| Please select the interest area of the program your child participated in?  (please select all that apply) | a. Aquatics (Swim Lessons/Training, Open Swim) b. Athletic (Basketball, Tennis, Pickleball, Football, Soccer) c. Cultural Events (Hikes & Tours, Historical Exploration, Field Trips) d. Education & Enrichment (Cooking/Baking, Computer Lab, Music Lessons) e. Fitness & Wellness Center f. Outdoor Recreation g. Dance (Ballet, Modern, Hip Hop) h. Afterschool Programs i. Social Activity (Arts and Crafts, Lego Building, etc.) j. Special Programs/Special Events k. Other [free text field] |
| How long was the program your child participated in? | a. One-time session b. 1-3 weeks c. 4-6 weeks d. 7-9 weeks e. > 10 weeks |
| What was the average amount of time spent at each session in the program? | a. Less than 1 hours  b. 1-3 hours  c. 3-6 hours  e. 6-8 hours  f. Full-day event |
| Did your child complete the entire registered program or at least half of the sessions? | a. Yes  b. No |
| Did your child participate in more than one program? | a. Yes  b. No |
| ***For participants that select Yes*** *transition back to the “Tell us about the program your child participated in”. Question sequence* | ***For participants that select No*** *transition to the “Tell us about your barriers to participation” section and then to the end of the survey* |
| **Tell us about your satisfaction with your local parks and recreation program** | |
| The registration process was easy to understand | a. Yes  b. No |
| **Please rate your satisfaction based on your participation while enrolled in the YES Study** | |
| The program matched our availability | Scale (Very Satisfied to Very Dissatisfied)   a. Very Satisfied b. Satisfied c. Neither Satisfied nor Dissatisfied d. Dissatisfied e. Very Dissatisfied |
| The program matched our budget | Scale (Very Satisfied to Very Dissatisfied)   a. Very Satisfied b. Satisfied c. Neither Satisfied nor Dissatisfied d. Dissatisfied e. Very Dissatisfied |
| I believe my child seemed to be in a better mood after participating in the program | Scale (Very Satisfied to Very Dissatisfied)   a. Very Satisfied b. Satisfied c. Neither Satisfied nor Dissatisfied d. Dissatisfied e. Very Dissatisfied |
| Overall, I am satisfied with my local parks and recreation program | Scale (Very Satisfied to Very Dissatisfied)   a. Very Satisfied b. Satisfied c. Neither Satisfied nor Dissatisfied d. Dissatisfied e. Very Dissatisfied |

| **Tell us about any barriers you faced during your participation in the YES Study that impacted your ability to register, reach or regularly attend the Parks and Recreation program(s)?**  *Please select all that apply* | |
| --- | --- |
|  | The registration process was not clear |
|  | Cleanliness and condition of recreation facilities |
|  | Safety and security of recreation areas |
|  | Time and distance required to travel to the local park and recreation center/program |
|  | Lack of transportation |
|  | Time constraints due to work, family, or other obligations |
|  | Availability of programs |
|  | I changed my mind and no longer wanted my child to participate in the study |
|  | The cost of participating in my local parks and recreation program did not align with my budget |
|  | Accessibility for people living with disabilities |
|  | My local park and recreation staff were unhelpful or unprofessional |
|  | I don't believe my child felt welcomed or included based on their identity |
|  | Availability of cell phone/Wi-Fi coverage |
|  | My child did not have friends participating in the program |
|  | General lack of interest or other priorities |
|  | Please share any other barriers you experienced: __________________ |
