# Supplementary material for: Youth Empowered Self-Care: a direct-to-participant intervention to promote mental well-being
Source: BMC Public Health. 2026 Jun 29;26:2403. doi: 10.1186/s12889-026-28143-0 (PMC13491775; doi:10.1186/s12889-026-28143-0)
Supplement: Supplementary file 5 [file 12889_2026_28143_MOESM5_ESM.docx]

# Supplemental Table 1. Tukey’s Post Hoc ANOVA Comparisons by Race

| **Dependent Variable** | **Pairwise Comparison** | **Mean Difference** | **Std. Error** | **p-value** | **95% CI** |
| --- | --- | --- | --- | --- | --- |
| Psychological Well-Being | Black – White | 1.67* | 0.41 | <.001 | [0.59, 2.74] |
| Psychological Well-Being | Black – Asian | 0.33 | 0.45 | 0.890 | [-0.85, 1.50] |
| Psychological Well-Being | Black – Other | 0.54 | 0.89 | 0.929 | [-1.76, 2.85] |
| Psychological Well-Being | White – Asian | -1.34* | 0.46 | 0.020 | [-2.53, -0.16] |
| Psychological Well-Being | White – Other | -1.13 | 0.89 | 0.587 | [-3.43, 1.18] |
| Psychological Well-Being | Asian – Other | 0.22 | 0.91 | 0.995 | [-2.14, 2.57] |
| Peer and Social Support | Black – White | 1.39* | 0.25 | <.001 | [0.73, 2.05] |
| Peer and Social Support | Black – Asian | 0.41 | 0.28 | 0.475 | [-0.32, 1.14] |
| Peer and Social Support | Black – Other | 0.61 | 0.55 | 0.690 | [-0.82, 2.04] |
| Peer and Social Support | White – Asian | -0.98* | 0.28 | 0.003 | [-1.71, -0.25] |
| Peer and Social Support | White – Other | -0.78 | 0.57 | 0.490 | [-2.21, 0.65] |
| Peer and Social Support | Asian – Other | 0.20 | 0.57 | 0.985 | [-1.26, 1.66] |
| Physical Well-Being | Black – White | 2.85* | 0.39 | <.001 | [1.83, 3.87] |
| Physical Well-Being | Black – Asian | 1.35* | 0.43 | 0.011 | [0.23, 2.47] |
| Physical Well-Being | Black – Other | 2.81* | 0.95 | 0.017 | [0.36, 5.26] |
| Physical Well-Being | White – Asian | -1.50* | 0.43 | 0.003 | [-2.62, -0.38] |
| Physical Well-Being | White – Other | -0.04 | 0.95 | 1.000 | [-2.41, 2.41] |
| Physical Well-Being | Asian – Other | 1.46 | 0.96 | 0.430 | [-1.04, 3.96] |
| Autonomy and Parent Relations | Black – White | 3.64* | 0.51 | <.001 | [2.32, 4.96] |
| Autonomy and Parent Relations | Black – Asian | 1.42 | 0.56 | 0.058 | [-0.03, 2.88] |
| Autonomy and Parent Relations | Black – Other | 3.03* | 1.10 | 0.033 | [0.18, 5.88] |
| Autonomy and Parent Relations | White – Asian | -2.22* | 0.56 | <.001 | [-3.68, -0.75] |
| Autonomy and Parent Relations | White – Other | -0.61 | 1.10 | 0.945 | [-3.47, 2.24] |
| Autonomy and Parent Relations | Asian – Other | 1.60 | 1.13 | 0.487 | [-1.32, 4.52] |
| School Environment | Black – White | 2.13* | 0.31 | <.001 | [1.33, 2.93] |
| School Environment | Black – Asian | 0.60 | 0.34 | 0.295 | [-0.28, 1.47] |
| School Environment | Black – Other | 1.21 | 0.67 | 0.271 | [-0.52, 2.94] |
| School Environment | White – Asian | -1.54* | 0.34 | <.001 | [-2.42, -0.65] |
| School Environment | White – Other | -0.92 | 0.67 | 0.517 | [-2.66, 0.81] |
| School Environment | Asian – Other | 0.61 | 0.68 | 0.806 | [-1.16, 2.39] |
| SCARED | Black – White | -2.96* | 0.71 | <.001 | [-4.80, -1.13] |
| SCARED | Black – Asian | -2.15* | 0.78 | 0.032 | [-4.16, -0.13] |
| SCARED | Black – Other | -4.88* | 1.54 | 0.009 | [-8.86, -0.90] |
| SCARED | White – Asian | -0.82 | 0.78 | 0.725 | [-1.21, 2.84] |
| SCARED | White – Other | -1.92 | 1.54 | 0.598 | [-5.91, 2.07] |
| SCARED | Asian – Other | -2.74 | 1.57 | 0.306 | [-6.81, 1.34] |

ANOVA = analysis of variance; SCARED = Screen for Child Anxiety Related Emotional Disorders.

# Supplemental Table 2. Tukey’s Post Hoc ANOVA Comparisons by Education

| **Dependent Variable** | **Pairwise Comparison** | **Mean Difference** | **Std. Error** | **p-value** | **95% CI** |
| --- | --- | --- | --- | --- | --- |
| Psychological Well-Being | HS Graduate– Some College | -.01 | .47 | 1.00 | [-1.31, 1.28] |
| Psychological Well-Being | HS Graduate– Bachelor’s | 1.52* | 0.39 | 0.001 | [0.44, 2.61] |
| Psychological Well-Being | HS Graduate– Graduate Degree | 2.31 | 1.15 | .262 | [-.84, 5.47] |
| Psychological Well-Being | Some College – Bachelor’s | 1.54* | 0.50 | 0.019 | [0.17, 2.91] |
| Psychological Well-Being | Some College – Graduate Degree | -2.33 | 1.19 | 0.289 | [-0.94, 5.59] |
| Psychological Well-Being | Bachelor’s – Graduate Degree | -0.79 | 1.16 | 0.961 | [-3.97, 2.40] |
| Peer and Social Support | HS Graduate– Some College | .44 | .29 | .525 | [-.34, 1.23] |
| Peer and Social Support | HS Graduate– Bachelor’s | 1.14 | .24 | <.001 | [.48, 1.80] |
| Peer and Social Support | HS Graduate– Graduate Degree | 3.54* | .70 | <.001 | [1.60, 5.47] |
| Peer and Social Support | Some College – Bachelor’s | .69 | .30 | .150 | [-.14, 1.52] |
| Peer and Social Support | Some College – Graduate Degree | 3.09* | .73 | <.001 | [1.09, 5.09] |
| Peer and Social Support | Bachelor’s – Graduate Degree | 2.40* | .71 | .008 | [.45, 4.35] |
| Physical Well-Being | HS Graduate– Some College | 1.28* | 0.44 | 0.035 | [0.06, 2.51] |
| Physical Well-Being | HS Graduate– Bachelor’s | 2.80* | 0.37 | <.001 | [1.77, 3.83] |
| Physical Well-Being | HS Graduate– Graduate Degree | 1.13 | 1.09 | 0.841 | [-1.88, 4.13] |
| Physical Well-Being | Some College – Bachelor’s | 1.52* | 0.47 | 0.012 | [.23, 2.80] |
| Physical Well-Being | Some College – Graduate Degree | -.16 | 1.13 | 1.00 | [-3.26, 2.95] |
| Physical Well-Being | Bachelor’s – Graduate Degree | -1.67 | 1.10 | 0.553 | [-4.70, 1.36] |
| Autonomy and Parent Relations | HS Graduate– Some College | 2.219* | 0.56 | <.001 | [0.69, 3.75] |
| Autonomy and Parent Relations | HS Graduate– Bachelor’s | 3.862* | 0.47 | <.001 | [2.56, 5.16] |
| Autonomy and Parent Relations | HS Graduate– Graduate Degree | 3.72 | 1.38 | 0.057 | [-.07, 7.51] |
| Autonomy and Parent Relations | Some College – Bachelor’s | -1.64* | 0.59 | 0.043 | [-0.03, 3.26] |
| Autonomy and Parent Relations | Some College – Graduate Degree | -1.50 | 1.42 | 0.829 | [-2.41, 5.41] |
| Autonomy and Parent Relations | Bachelor’s – Graduate Degree | -.14 | 1.39 | 1.000 | [-3.96, 3.68] |
| School Environment | HS Graduate– Some College | .55 | .34 | .492 | [-.39, 1.49] |
| School Environment | HS Graduate– Bachelor’s | 2.21* | 0.29 | <.001 | [1.41, 3.01] |
| School Environment | HS Graduate– Graduate Degree | 1.67 | 0.85 | 0.289 | [-.67, 4.01] |
| School Environment | Some College – Bachelor’s | 1.66 | 0.36 | <.001 | [.65, 2.66] |
| School Environment | Some College – Graduate Degree | 1.12 | 0.88 | 0.708 | [-1.30, 3.54] |
| School Environment | Bachelor’s – Graduate Degree | -0.54 | 0.86 | 0.971 | [-2.91, 1.83] |
| SCARED | HS Graduate– Some College | -.85 | .78 | .811 | [-.2.98, 1.29] |
| SCARED | HS Graduate– Bachelor’s | -3.56* | .66 | <.001 | [-5.37, -1.75] |
| SCARED | HS Graduate– Graduate Degree | .03 | 1.93 | 1.00 | [-5.29, 5.35] |
| SCARED | Some College – Bachelor’s | -2.71* | .82 | .010 | [-4.98, -.44] |
| SCARED | Some College – Graduate Degree | .88 | 2.00 | .992 | [-4.62, 6.37] |
| SCARED | Bachelor’s – Graduate Degree | 3.59 | 1.95 | .356 | [-1.79, 8.96] |

ANOVA = analysis of variance; SCARED = Screen for Child Anxiety Related Emotional Disorders.

# Supplemental Table 3. Tukey’s Post Hoc ANOVA Comparisons by Household Income

| **Dependent Variable** | **Pairwise Comparison** | **Mean Difference** | **Std. Error** | **p-value** | **95% CI** |
| --- | --- | --- | --- | --- | --- |
| Psychological Well-Being | $25K–50K vs $50–75K | 1.27 | 0.93 | 0.648 | [-1.28, 3.81] |
| Psychological Well-Being | $25K–50K vs $75–100K | 2.26 | 0.93 | 0.111 | [-0.30, 4.83] |
| Psychological Well-Being | $25K–50K vs >$100K | 2.81^*^ | 0.99 | 0.039 | [0.09, 5.53] |
| Psychological Well-Being | $25K–50K vs Prefer not answer | 0.42 | 1.22 | 0.997 | [-2.93, 3.77] |
| Psychological Well-Being | $50–75K vs $75–100K | 1.00 | 0.39 | 0.088 | [-0.09, 2.08] |
| Psychological Well-Being | $50–75K vs >$100K | 1.54^*^ | 0.51 | 0.024 | [0.13, 2.95] |
| Psychological Well-Being | $50–75K vs Prefer not answer | -0.85 | 0.88 | 0.869 | [-3.27, 1.56] |
| Psychological Well-Being | $75–100K vs >$100K | 0.55 | 0.52 | 0.833 | [-0.89, 1.99] |
| Psychological Well-Being | $75–100K vs Prefer not answer | -1.85 | 0.88 | 0.228 | [-4.28, .58] |
| Psychological Well-Being | >$100K vs Prefer not answer | -2.39 | 0.94 | 0.09 | [-4.99, .20] |
| Peer and Social Support | $25K–50K vs $50–75K | 0.86 | 0.57 | 0.562 | [-0.71, 2.43] |
| Peer and Social Support | $25K–50K vs $75–100K | 1.31 | 0.57 | 0.155 | [-0.27, 2.89] |
| Peer and Social Support | $25K–50K vs >$100K | 2.41^*^ | 0.61 | 0.001 | [0.73, 4.08] |
| Peer and Social Support | $25K–50K vs Prefer not answer | -0.29 | 0.75 | 0.995 | [-2.36, 1.78] |
| Peer and Social Support | $50–75K vs $75–100K | 0.45 | 0.24 | 0.328 | [-0.21, 1.11] |
| Peer and Social Support | $50–75K vs >$100K | 1.55^*^ | 0.32 | <.001 | [0.68, 2.42] |
| Peer and Social Support | $50–75K vs Prefer not answer | -1.15 | 0.54 | 0.214 | [-2.64, .34] |
| Peer and Social Support | $75–100K vs >$100K | 1.09^*^ | 0.32 | 0.007 | [0.21, 1.98] |
| Peer and Social Support | $75–100K vs Prefer not answer | -1.60^*^ | 0.54 | 0.030 | [-3.10, -0.10] |
| Peer and Social Support | >$100K vs Prefer not answer | -2.70^*^ | 0.58 | <.001 | [-4.30, -1.10] |
| Physical Well-Being | $25K–50K vs $50–75K | 3.38^*^ | 0.89 | 0.002 | [0.93, 5.83] |
| Physical Well-Being | $25K–50K vs $75–100K | 4.37^*^ | 0.897 | <.001 | [1.91, 6.82] |
| Physical Well-Being | $25K–50K vs >$100K | 5.70^*^ | 0.95 | <.001 | [3.10, 8.31] |
| Physical Well-Being | $25K–50K vs Prefer not answer | 1.95 | 1.14 | 0.430 | [-1.19, 5.09] |
| Physical Well-Being | $50–75K vs $75–100K | 0.99 | 0.38 | 0.072 | [-0.05, 2.03] |
| Physical Well-Being | $50–75K vs >$100K | 2.33^*^ | 0.49 | <.001 | [0.97, 3.68] |
| Physical Well-Being | $50–75K vs Prefer not answer | -1.43 | 0.80 | 0.39 | [-3.64, .78] |
| Physical Well-Being | $75–100K vs >$100K | 1.34 | 0.50 | 0.061 | [-0.04, 2.71] |
| Physical Well-Being | $75–100K vs Prefer not answer | -2.42^*^ | 0.81 | 0.026 | [-4.64, -0.19] |
| Physical Well-Being | >$100K vs Prefer not answer | -3.75^*^ | 0.87 | <.001 | [-6.14, -1.37] |
| Autonomy and Parent Relations | $25K–50K vs $50–75K | 2.47 | 1.26 | 0.29 | [-0.99, 5.93] |
| Autonomy and Parent Relations | $25K–50K vs $75–100K | 3.97^*^ | 1.26 | 0.016 | [0.50, 7.45] |
| Autonomy and Parent Relations | $25K–50K vs >$100K | 5.59^*^ | 1.34 | <.001 | [1.89, 9.22] |
| Autonomy and Parent Relations | $25K–50K vs Prefer not answer | 0.89 | 1.58 | 0.980 | [-3.46, 5.23] |
| Autonomy and Parent Relations | $50–75K vs $75–100K | 1.51^*^ | 0.50 | 0.024 | [0.13, 2.88] |
| Autonomy and Parent Relations | $50–75K vs >$100K | 3.09^*^ | 0.65 | <.001 | [1.29, 4.89] |
| Autonomy and Parent Relations | $50–75K vs Prefer not answer | -1.58 | 1.07 | 0.577 | [-4.52, 1.36] |
| Autonomy and Parent Relations | $75–100K vs >$100K | 1.58 | 0.67 | 0.126 | [-0.25, 3.42] |
| Autonomy and Parent Relations | $75–100K vs Prefer not answer | -3.09 | 1.08 | 0.036 | [-6.05, -.13] |
| Autonomy and Parent Relations | >$100K vs Prefer not answer | -4.67^*^ | 1.16 | 0.001 | [-7.85, -1.49] |
| School Environment | $25K–50K vs $50–75K | 1.39 | 0.70 | 0.282 | [-0.55, 3.33] |
| School Environment | $25K–50K vs $75–100K | 2.08* | 0.71 | .030 | [-0.55, 3.33] |
| School Environment | $25K–50K vs >$100K | 3.32* | 0.75 | <.001 | [1.25, 5.39] |
| School Environment | $25K–50K vs Prefer not answer | -0.28 | 0.91 | 0.998 | [-2.77, 2.22] |
| School Environment | $50–75K vs $75–100K | 0.69 | 0.30 | 0.143 | [-0.13,1.51] |
| School Environment | $50–75K vs >$100K | 1.93^*^ | 0.39 | <.001 | [0.86, 3.00] |
| School Environment | $50–75K vs Prefer not answer | -1.67 | 0.64 | 0.071 | [-3.42,.08] |
| School Environment | $75–100K vs >$100K | 1.24^*^ | 0.40 | 0.017 | [0.15, 2.34] |
| School Environment | $75–100K vs Prefer not answer | -2.35^*^ | 0.64 | 0.003 | [-4.12, -0.59] |
| School Environment | >$100K vs Prefer not answer | -3.60^*^ | 0.69 | <.001 | [-5.49, -1.70] |
| SCARED | $25K–50K vs $50–75K | -3.73 | 1.54 | 0.113 | [-7.97, 0.50] |
| SCARED | $25K–50K vs $75–100K | -5.70^*^ | 1.55 | 0.003 | [-9.96, -1.43] |
| SCARED | $25K–50K vs >$100K | -7.60^*^ | 1.64 | <.001 | [-12.13, -3.08] |
| SCARED | $25K–50K vs Prefer not answer | -0.57 | 1.98 | 0.998 | [-6.02, 4.87] |
| SCARED | $50–75K vs $75–100K | -1.96^*^ | 0.65 | 0.022 | [-3.74, -0.19] |
| SCARED | $50–75K vs >$100K | -3.87^*^ | 0.85 | <.001 | [-6.21, -1.54] |
| SCARED | $50–75K vs Prefer not answer | 3.16 | 1.39 | 0.16 | [-0.67, 6.98] |
| SCARED | $75–100K vs >$100K | -1.91 | 0.87 | 0.183 | [-4.29, 0.48] |
| SCARED | $75–100K vs Prefer not answer | 5.12^*^ | 1.40 | 0.003 | [1.26, 8.98] |
| SCARED | >$100K vs Prefer not answer | 7.03^*^ | 1.51 | <.001 | [2.88, 11.17] |

ANOVA = analysis of variance; SCARED = Screen for Child Anxiety Related Emotional Disorders.
