# Supplementary material for: Youth Empowered Self-Care: a direct-to-participant intervention to promote mental well-being
Source: BMC Public Health. 2026 Jun 29;26:2403. doi: 10.1186/s12889-026-28143-0 (PMC13491775; doi:10.1186/s12889-026-28143-0)
Supplement: Supplementary file 6 [file 12889_2026_28143_MOESM2_ESM.docx]

YES Pilot Study Design Flow
